# Neutrophil Extracellular Trap Formation Is Associated with IL-1β and Autophagy-Related Signaling in Gout

**DOI:** 10.1371/journal.pone.0029318

**Published:** 2011-12-16

**Authors:** Ioannis Mitroulis, Konstantinos Kambas, Akrivi Chrysanthopoulou, Panagiotis Skendros, Eirini Apostolidou, Ioannis Kourtzelis, Georgios I. Drosos, Dimitrios T. Boumpas, Konstantinos Ritis

**Affiliations:** 1 First Department of Internal Medicine, Democritus University of Thrace, University General Hospital of Alexandroupolis, Alexandroupolis, Greece; 2 Department of Orthopaedic Surgery, Democritus University of Thrace, University General Hospital of Alexandroupolis, Alexandroupolis, Greece; 3 Department of Rheumatology, Clinical Immunology and Allergy, University Hospital, Medical School, University of Crete, Heraklion, Greece; McGill University, Canada

## Abstract

**Background:**

Gout is a prevalent inflammatory arthritis affecting 1–2% of adults characterized by activation of innate immune cells by monosodium urate (MSU) crystals resulting in the secretion of interleukin-1β (IL-1β). Since neutrophils play a major role in gout we sought to determine whether their activation may involve the formation of proinflammatory neutrophil extracellular traps (NETs) in relation to autophagy and IL-1β.

**Methodology/Principal Findings:**

Synovial fluid neutrophils from six patients with gout crisis and peripheral blood neutrophils from six patients with acute gout and six control subjects were isolated. MSU crystals, as well as synovial fluid or serum obtained from patients with acute gout, were used for the treatment of control neutrophils. NET formation was assessed using immunofluorescence microscopy. MSU crystals or synovial fluid or serum from patients induced NET formation in control neutrophils. Importantly, NET production was observed in neutrophils isolated from synovial fluid or peripheral blood from patients with acute gout. NETs contained the alarmin high mobility group box 1 (HMGB1) supporting their pro-inflammatory potential. Inhibition of phosphatidylinositol 3-kinase signaling or phagolysosomal fusion prevented NET formation, implicating autophagy in this process. NET formation was driven at least in part by IL-1β as demonstrated by experiments involving IL-1β and its inhibitor anakinra.

**Conclusions/Significance:**

These findings document for the first time that activation of neutrophils in gout is associated with the formation of proinflammatory NETs and links this process to both autophagy and IL-1β. Modulation of the autophagic machinery may represent an additional therapeutic study in crystalline arthritides.

## Introduction

Acute gout is a prevalent inflammatory arthritis that results from monosodium urate (MSU) crystal deposition. It affects up to 1–2% of adults and is the most common inflammatory arthritis in men [1]. MSU crystals are endogenous danger signals, which activate articular resident cells of the monocyte/macrophage lineage, resulting in the triggering of inflammatory attacks [2], [3], [4]. Even though several proinflammatory cytokines and chemokines have been associated with the early phase of acute gouty arthritis, growing evidence derived from experimental and clinical studies indicates a pivotal role for interleukin-1β (IL-1β) in the initiation of inflammation. Activation of NLRP3 inflammasome by MSU crystals is thought to regulate pro-IL-1β processing during gout [5], [6]. Moreover, neutrophil-derived proteases have been reported to contribute to IL-1β production [7]. Inhibition of IL-1β signaling is effective in the resolution of gouty inflammation in both animal models [8], [9] and in humans [10]–[13].

In gout, initial activation of articular cells by MSU crystals is followed by the recruitment and ingress of large numbers of neutrophils into the inflamed joints [14]. *In vitro* studies have previously attempted to elucidate the mechanism that drives neutrophil activation by MSU-crystals and proposed the stimulation of several kinases including Src-family tyrosine kinase [15], protein kinase C [16] and phosphatidylinositol 3-kinases (PI3Ks) [17], [18] as key signaling events in this process.

PI3K signaling has been previously implicated in the initiation of autophagy in human neutrophils in response to several inflammatory stimuli [19]. Autophagy constitutes a critical cellular mechanism for the preservation of cell integrity, while it is implicated in the regulation of innate immune functions [20]. Recent data suggest that autophagy is required for NETosis, a distinct form of neutrophil cell death, characterized by the release of neutrophil extracellular traps (NETs) [21].

NETs are extracellular fibrous structures composed of chromatin and granule constituents of neutrophils [22]. NET formation after phagocytosis of pathogens or treatment with inflammatory stimuli has been recently described as an extracellular antimicrobial process, critical for neutrophil physiology [22]. It is suggested that capture and killing of microbes by the formation of NETs constitutes an additional mechanism for pathogen elimination which expands neutrophil microbicidal activity after cell death [22], [23]. However, NET release from cells not undergoing NETosis has also been reported [24]. The localization of several neutrophil enzymes with proinflammatory function, like elastase, myeloperoxidase (MPO) or proteinase 3, to NETs and the increasing evidence for the implication of NETs in non-infectious diseases, including asthma [25], ulcerative colitis [26] and systemic lupus erythematosus (SLE) [27]–[29], suggest a role for the formation of these structures in the amplification of the inflammatory responses that characterize these disorders.

Since both neutrophils and IL1β play a major role in the pathogenesis of acute gout, we studied the generation of NETs during acute gout and its relation to autophagy and IL-1β. Herein, we report for the first time proinflammatory NET formation from neutrophils derived from synovial fluid and peripheral neutrophils from patients with acute gout and control neutrophils stimulated with MSU crystals. We also present data linking this to autophagy and IL-1β.

## Results

### MSU crystals induce the formation of NETs

**Figure 1:**
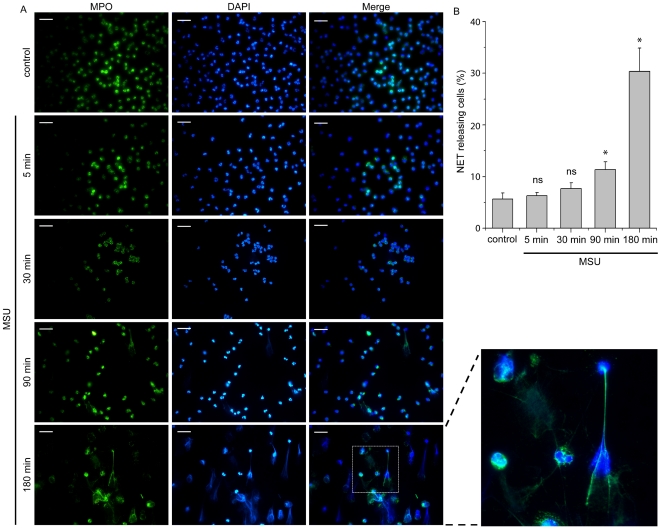
PMNs treated with MSU crystals release NETs. PMNs from control subjects treated with MSU crystals for different time points. NETs are assessed by immunofluorescence microscopy after co-staining with DAPI (blue) and MPO (green). Control represents untreated PMNs incubated for 180 min. Scale bars, 30 µm. Original magnification 400x. One out of three independent experiments is shown. B) Percentage of NET releasing cells presented in panel A. Data are representative of three independent experiments and presented as mean ± SD. * *P*<0.05; *ns* = non significant compared to control cells.

**Figure 2:**
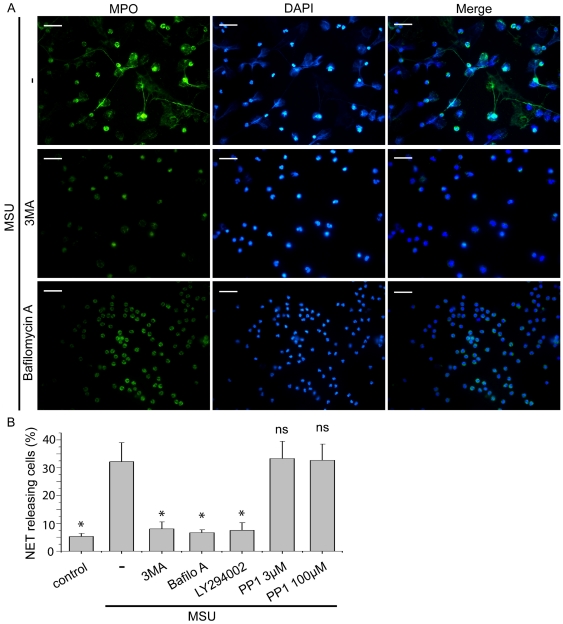
PI3K signaling and endosomal acidification is involved in NET release after treatment with MSU crystals. A) Treatment with 3-MA or bafilomycin A prevents the release of NETs from PMNs from control subjects treated with MSU crystals for 180 min. NETs are determined by co-staining with DAPI (blue) and MPO (green) and visualized by immunofluorescence microscopy. Scale bars, 30 µm. Original magnification 400x. One out of four independent experiments is shown. B) The effect of treatment with 3-MA, LY294002, bafilomycin A or PP1 in the percentage of NET releasing PMNs incubated with MSU crystals. Data are representative of four independent experiments and presented as mean ± SD. * *P*<0.05; *ns* = non significant compared to MSU treated cells.

The ability of MSU crystals to induce neutrophil activation and NET release was initially studied by treating peripheral polymorphonuclear cells (PMNs) with MSU crystals for 5 min, 30 min, 90 min and 3 h. The presence of web-like structures, visualized by co-staining with diamidino-2-phenylindole (DAPI) and MPO, was observed after 90 min of treatment and was prominent at 3 h (Fig. 1), indicating the formation of NETs [23]. To examine the possible implication of the autophagic machinery in the release of these structures, 3-methyladenine (3-MA), an inhibitor of class III phosphatidylinositol 3-kinase (PI3K) signaling [19], LY294002 (Fig. S1), a pan-PI3K inhibitor, or bafilomycin A, which prevents autophagosome maturation by inhibiting their fusion with lysosomes [30], were used. Treatment of PMNs with these agents 30 min after the addition of MSU crystals significantly diminished the percentage of cells releasing the aforementioned structures (Fig. 2). On the other hand, Src kinase inhibition had no effect on NET formation (Fig 2B, Fig. S1).

**Figure 3:**
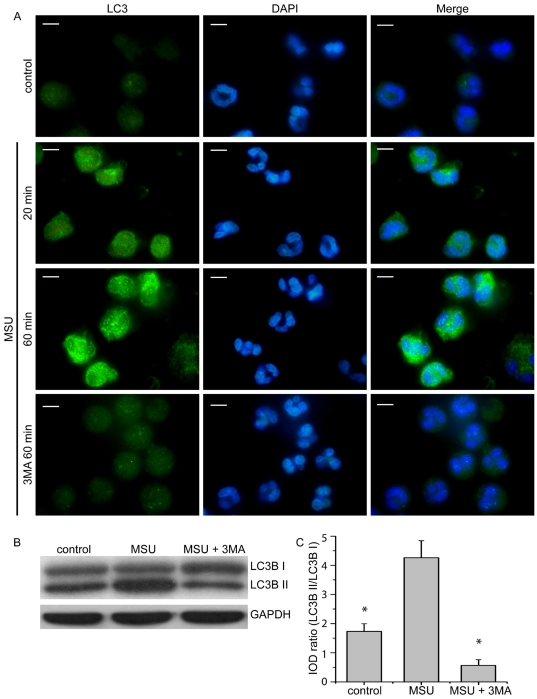
Induction of autophagy in PMNs treated with MSU crystals. A) Induction of autophagy in control PMNs treated with MSU crystals for 20 min and 60 min, as assessed by immunofluorescence microscopy of endogenous LC3B. LC3B-positive autophagosome formation was blocked by 3-MA. DNA is labeled with DAPI (blue). LC3B was stained with polyclonal anti-LC3B Ab (green). Scale bars, 5 µm. Original magnification 1000x. One out of three independent experiments is shown. B) Induction of LC3B-I to LC3B –II conversion in PMNs treated with MSU crystals for 45 min, as assessed by immunoblotting. Attenuation of LC3B conversion in PMNs treated with 3-MA. C) Measurement of integrated optical density (IOD) of bands presented in B, expressed as LC3B-II to LC3B-I ratio ± SD. Representative data from four independent experiments are shown in B and C. * *P*<0.05 compared to MSU treated cells.

Moreover, the activation of autophagy in PMNs treated with MSU crystals was assessed by endogenous LC3B immunofluorescence. LC3B punctuated structures, associated with autophagy induction, were observed in cells treated for both selected time points, 20 min and 60 min. This effect was inhibited by 3-MA (Fig. 3A). The ability of MSU crystals to induce autophagy was also demonstrated by LC3B immunoblotting of cell lysates from PMNs treated for 45 min (Fig. 3B and C). Additionally, treatment of PMNs with MSU crystals for 3 h did not induce apoptosis or necrosis (Fig. S3B).

### Release of extracellular DNA traps by synovial fluid neutrophils from patients with acute gout

**Figure 4:**
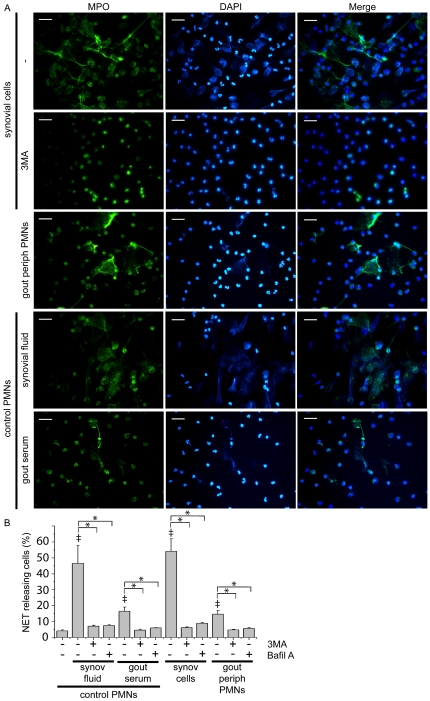
NET release from synovial cells and peripheral PMNs from patients with gout. A) NET formation by synovial cells, peripheral PMNs from patients with gout and from control PMNs treated with synovial fluid or serum from patients with gout, as assessed by immunofluorescense microscopy after co-staining with DAPI (blue) and MPO (green). Inhibitory effect of treatment with 3-MA on the release of NETs from synovial cells. Scale bars, 30 µm. Original magnification 400x. One out of six independent experiments is shown. B) The effect of treatment with 3-MA or bafilomycin A in the percentage of NET releasing cells presented in panel A. Data are representative of six independent experiments and presented as mean ± SD. ‡ *P*<0.05 compared to control, * *P*<0.05.

We further studied whether cells isolated from inflamed joints from patients with gout could release extracellular fibrous formations. Formation of extracellular structures, visualized by staining with DAPI and MPO, by synovial fluid cells was observed after 3 h of incubation (Fig. 4, Fig. S2). Considering that neutrophils were the predominant cell population in synovial fluid (>95%), as demonstrated by May-Grunwald staining (Fig. S3A), we conclude that this cell type was responsible for this observation. To explore whether the inflammatory environment of gout induces the formation of such structures, control PMNs were treated with synovial fluid derived from inflamed joints. Extensive NET release was observed under these conditions (Fig. 4), while treatment with 3-MA or bafilomycin A abrogated this effect (Fig. 4B).

### NET formation by peripheral PMNs from patients with acute gout

We next investigated whether NET production is a restricted to affected joints response that depends on the localized activation of PMNs by MSU crystals or a systemic phenomenon in the context of gout. Peripheral PMNs from patients with gout released NETs, determined by co-staining with MPO and DAPI, when incubated for 3 h in a less prominent way compared to those observed in synovial fluid cells (Fig. 4). Moreover, treatment of control PMNs with serum from patients with acute gout had minimal but statistically significant effect (Fig. 4). PI3K inhibition or treatment with bafilomycin A effectively blocked this effect (Fig 4B).

### IL-1β blockade attenuates the effect of gouty synovial fluid on NET formation

**Figure 5:**
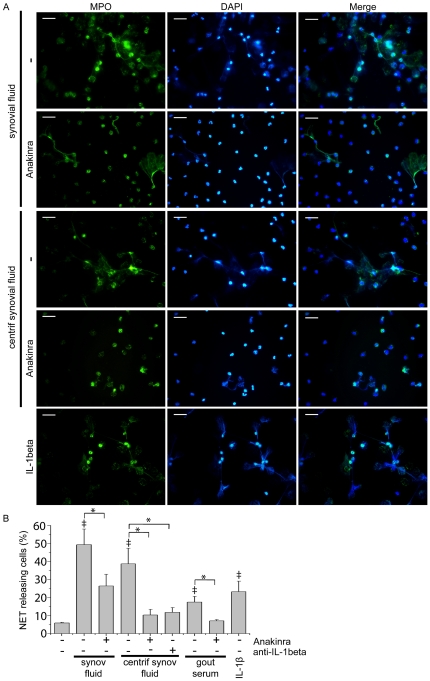
Implication of IL-1β in the induction of NET release from PMNs treated with synovial fluid or serum from patients with gout. A) The inhibitory effect of treatment with anakinra on NET release from control PMNs treated with centrifuged or not synovial fluid from patients with gout, as assessed by immunofluorescense microscopy after co-staining with DAPI (blue) and MPO (green). Treatment with recombinant IL-1β induces NET release. Scale bars, 30 µm. Original magnification 400x. One out of six independent experiments is shown. B) The inhibitory effect of anakinra on the percentage of NET releasing PMNs presented in panel A and in PMNs treated with serum from patients with gout. Incubation with anti-IL-1β mAb reduced the percentage of NET releasing PMNs treated with centrifuged synovial fluid. Data are representative of six independent experiments and presented as mean ± SD. ‡ *P*<0.05 compared to control, * *P*<0.05.

Given the previously suggested critical role of IL-1β in the pathogenesis of gout [6], we studied the possible effect of inhibition of IL-1 signaling on NET formation. Treatment with anakinra, a recombinant IL-1 receptor antagonist, partially inhibited NET production, determined by co-staining with MPO and DAPI, from control PMNs treated with synovial fluid from patient with gout (Fig 5). To further implicate IL-1 in this process, synovial fluid was centrifuged, in order to reduce MSU crystal concentration. IL-1 signaling inhibition attenuated the observed NET release from control neutrophils treated with centrifuged synovial fluid (Fig 5A and B). Moreover, anakinra completely inhibited the respective effect of serum derived from patients with acute gout on NET production from control PMNs (Fig 5B). To discriminate whether the effect of anakinra was due to the inhibition of IL-1a or IL-1β signaling, centrifuged synovial fluid was treated with anti-IL-1β monoclonal antibody (mAb) prior to stimulation of control PMNs (Fig 5B). Both anti-IL-1β and anakinra treatment had a similar inhibitory effect. Moreover, incubation of control PMNs with recombinant IL-1β induced the formation of NETs (Fig 5).

### Extracellular DNA structures from synovial fluid neutrophils are decorated with high mobility group box chromosomal protein 1 (HMGB1)

**Figure 6:**
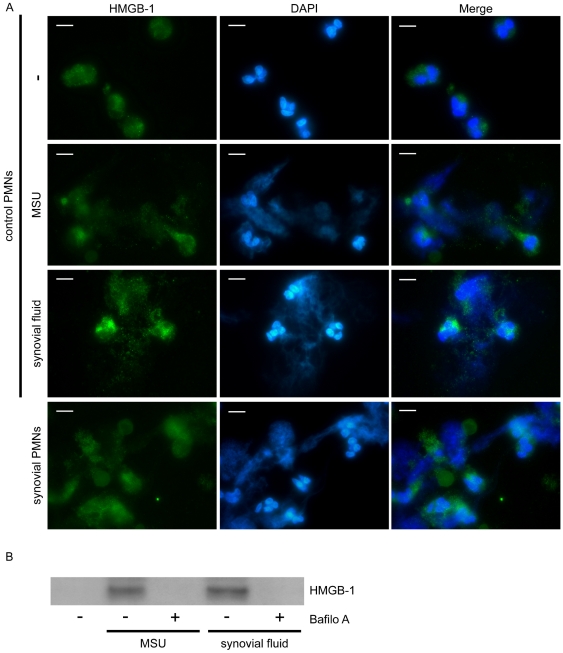
Localization of HMGB-1 on extracellular fibrous DNA structures. A) Expression of HMGB-1 in extracellular DNA structures released from PMNs derived from control subjects treated with MSU crystals or synovial fluid and synovial cells from patients with gout, as assessed by immunofluorescense microscopy. DNA is labeled with DAPI (blue). HMGB-1 is stained with anti-HMGB-1 mAb (green). Control represents untreated PMNs. One representative out of four independent experiments is shown. Scale bars, 5 µm. Original magnification 1000x. B) Expression of HMGB-1 in NETs released from control PMNs stimulated with MSU crystals or synovial fluid from patients with gout, as assessed by immunoblotting of NET-derived proteins. The inhibitory effect of bafilomycin A is shown. One out of three independent experiments is shown.

Given that HMGB1, an alarmin that activates the innate immune system [31], has been recently identified in large amounts on NETs from patients with pediatric SLE [27], its localization on extracellular DNA web-like formations, assessed by staining with DAPI, released by synovial fluid PMNs from patients with acute gout was investigated. We observed that HMGB1 was localized in the aforementioned extracellular structures released from synovial fluid PMNs (Fig. 6) or control PMNs treated with either MSU crystals or synovial fluid from patients with gout (Fig. 6).

The expression of HMGB1 on extracellular DNA structures released by control PMNs treated with MSU crystals or synovial fluid was also demonstrated by immunoblotting of proteins isolated from these structures. Treatment with bafilomycin A abrogated this effect (Fig. 6).

## Discussion

In this report, we provide for the first time evidence for the formation of NETs during acute gout. Our experimental data indicate that inhibition of either phagolysosomal fusion or PI3K signaling hindered NET release from control neutrophils treated with MSU crystals. Peripheral and synovial fluid neutrophils derived from patients with gouty inflammation formed NETs in an autophagy-dependent manner while synovial fluid from affected joints and serum derived from patients with gout induced NET release by control neutrophils. We also provide evidence that IL-1β signaling is implicated, at least in part, in the formation of NETs, as shown by the inhibitory effect of anakinra and anti-IL-1β mAb.

Neutrophils are thought to represent critical effector cells responsible for gouty inflammation [14]. Neutrophil enzymes have been previously proposed to participate in the cleavage of pro-IL-1β in a MSU-driven model of inflammation [7]. Considering that the release of these enzymes promotes tissue injury and inflammation and since NET formation results in the extracellular exposure of proteolytic enzymes in the inflamed tissues, a key role for NET release in the induction of inflammation during the context of acute gout is suggested. Moreover, the identification of HMGB1 in NETs released from synovial fluid neutrophils argues for the pro-inflammatory potential of these extracellular structures. HMGB1 is a nuclear protein that acts as an inflammatory mediator for the innate immune system when released to the extracellular milieu [31]. Even though it has not been directly involved in the pathogenesis of gout, HMGB1 is implicated in the pathomechanism of rheumatoid [32] and experimental arthritis [33], suggesting a possible role in the induction of gouty arthritis.

In this study, 3-MA and LY 294002 were utilized as PI3K inhibitors and bafilomycin A as an inhibitor of endosomal acidification. Among other functions, PI3K signaling is crucial in the initiation of autophagy while early autophagosome fusion with lysosomes and acidification of autophagosomes constitute a terminal event in this process [20]. In view of the inhibitory effect of 3-MA and bafilomycin A on NET formation, we propose that intact autophagic machinery is required for the release of these extracellular structures. This is in accordance with a recent report demonstrating that the inhibition of autophagy in neutrophils treated with phorbol myristate acetate prevented NETosis and led to apoptotic cell death [21]. However, the interplay between neutrophil autophagic machinery and NET formation in the pathogenesis of an inflammatory disorder has not been previously described. Our findings are in accordance with the previously described involvement of MSU-containing phagolysosomal formation [34] and PI3K signaling [17], [18] in the activation of neutrophils by MSU crystals. In contrast to previous studies which demonstrated the implication of Src kinase signaling in the activation of neutrophils [15], no such correlation was proven in our model.

NET formation was further associated with IL-1 signaling. Anakinra partially inhibited NET release from PMNs treated with synovial fluid derived from patients with active arthritis. When MSU crystal burden was minimized by centrifugation, the inhibitory effect of anakinra became more obvious, implying that IL-1 signaling acts in parallel with a possible direct effect of MSU crystals. Whether the previously reported role of IL-1β in the induction of autophagy in human neutrophils [19] is responsible for this effect cannot be confirmed due to the uniform inhibitory effect of autophagy inhibition on NET formation. Our data provide novel insight in the role of IL-1β in the inflammatory milieu of gout. Concerning the pathogenesis of the disorder, IL-1β is considered a pivotal cytokine [6]. In addition to the ability of MSU crystals to induce pro-IL-1β proteolytic cleavage *via* NLRP3 activation [5], studies in animal models suggest that this cytokine participates in the neutrophil chemotaxis and influx into the site of inflammation [8], [9], while animals deficient in critical components of IL-1 signaling were resistant to MSU induced inflammation [8]. The critical role of IL-1β in the pathogenesis of gout was confirmed by the beneficial clinical outcome of IL-1β blockade in patients with gout [10]–[13].

In conclusion, our data indicate that NETs are formed during inflammatory attacks of gout and suggest a linkage between NET release and signaling involved in autophagy and IL-1 activity. Our study provides a springboard for further investigation of NET release in the pathogenesis of inflammatory arthritis. Modulation of the autophagic machinery may represent an additional therapeutic target in crystalline arthritis.

## Materials and Methods

### Patients

Synovial fluid was collected from six patients with active gout attack, centrifuged at 800x g to separate cells from supernatant. Supernatants were stored at −20°C until used. To minimize the concentration of MSU crystals, synovial fluid was centrifuged at 12000x g for 15 min. A decrease in MSU crystal concentration greater that 70% was observed after centrifugation, as observed by microscopy. Synovial fluid cell populations were characterized by May-Grunwald and indicated a neutrophil predominance (>90%). Synovial cells were used immediately for determination of NET formation. Furthermore, PMNs were isolated from heparinized blood from six patients and six healthy donors by Histopaque double-gradient density centrifugation as previously described [35]. Serum was also isolated from patients with gout. The study protocol design was in accordance of the Declaration of Helsinki and the procedures have been approved by the local ethics committee (Scientific Committee of the University Hospital of Alexandroupolis, Greece). Informed, written consent has been obtained from all participants involved in the present study.

### Reagents

Uric acid sodium salt, human recombinant IL-1β, bafilomycin A, PP1, 4′,6-Diamidino-2-phenylindole (DAPI), *Limulus* amebocyte assay and anti-LC3B polyclonal Ab were purchased from Sigma-Aldrich (St. Louis, MO). 3-MA and LY 294002 were purchased from Calbiochem (San Diego, CA), IL-1β mouse mAb and HRP-conjugated secondary antibody from R&D Systems (Minneapolis, MN), MPO-specific mouse mAb from DAKO (Denmark); HMGB1-specific mouse mAb and GAPDH polyclonal Ab from Abcam (UK), polyclonal rabbit anti-mouse Alexa Fluor 488 Ab and polyclonal goat anti-rabbit Alexa Fluor 488 Ab from Invitrogen (Carlsbad, CA). In experiments using pharmacological inhibitors, control PMNs were treated with an equivalent concentration of vehicle (0.1% DMSO).

### MSU crystal preparation

MSU crystals were prepared under pyrogen-free conditions. Urate acid sodium salt (Sigma-Aldrich) was dissolved in 1 M NaOH (25 mg/ml) and boiled for 2 hours at 200°C prior to crystallization. The solution was left to cool at room temperature and filtered through a 0.2 µM filter. It was then incubated at room temperature for 7 days. The resulting crystals were washed with ethanol and acetone and allowed to air dry under sterile conditions. Triclinic MSU crystals were needle-shaped, between 5 and 20 µm in length and verified to be free of detectable LPS contamination by the *Limulus* amebocyte cell lysate assay (Sigma-Aldrich).

### Stimulation and inhibition studies

Neutrophils or synovial fluid cells were incubated at 37°C in a total volume of 500 µl of RPMI (Gibco BRL, Gaithersburg, MD) in the presence of 2% serum from healthy donor and different stimulatory agents. In the set of experiments evaluating the effect of treatment with MSU crystals, neutrophils were treated for 5 min, 30 min, 90 min and 3 h. To study autophagy induction, neutrophils were treated for 20, 45 or 60 min [19], [21]. Neutrophils were treated with MSU crystals (250 µg/ml) or synovial fluid supernatant (20 µl) or serum from patients with gout (20 µl). The doses were selected according to optimization experiments. Cells were also stimulated with human recombinant IL-1β (100 ng/ml) [19]. To inhibit the autophagic machinery, cells were treated with the PI3K inhibitor 3-MA (5 mM) [19] or LY 294002 (50 µM) [19], or bafilomycin A (30 nM) [36] for the inhibition of autophagolysosomal fusion after 30 min of stimulation in order to permit phagocytosis of crystals to take place. For Src kinase inhibition the selective inhibitor PP1 (3 and 100 µM) was used. IL-1β inhibition was performed by Anakinra (Kineret; Amgen/Biovitrum AB) at a concentration of 100 ng/ml, according to neutralizing optimization experiments, or a neutralizing IL-1β mouse monoclonal antibody (10 µg/ml), according to manufacturer's instructions. All experiments were performed in triplicates. Viability of cells treated with MSU for 3 h was assessed by flow cytometry using propidium iodide (PI, Sigma-Aldrich) to stain necrotic cells and an antibody against Annexin-V (BD Biosciences) as an apoptotic marker. Cells were analyzed in a FACScan flow cytometer (BD Biosciences). All the materials in this study were endotoxin free, as determined by a *Limulus* amebocyte assay.

### Immunofluorescence

To visualize NET formation, isolated neutrophils or synovial fluid cells were seeded in lysine-coated glass coverslips and prepared as previously described [37]. In brief, cells, after incubation, were fixed in 4% paraformaldehyde for 2–4 hours at room temperature. All samples were prepared in triplicates. Nonspecific binding sites were blocked with 5% rabbit serum in PBS. NETs were stained using an MPO-specific mouse mAb or an HMGB-1-specific mouse mAb. A polyclonal rabbit anti-mouse Alexa Fluor 488 antibody was utilized as secondary. DNA was counterstained using DAPI. Cell preparations were visualized in a fluorescence microscope (Leica DM2000). The percentage of cells undergoing NET release was determined by examining 200 cells per sample in a double blind fashion.

For LC3B immunofluorescence, neutrophils were prepared as described above and were stained with an anti-LC3B polyclonal Ab, followed by a polyclonal goat anti-rabbit Alexa Fluor 488 antibody utilized as secondary. Cells were counterstained using DAPI.

### Protein purification from NETs

NET protein purification was performed as previously described [38]. In brief, neutrophils were seeded in 6-well culture plates (Corning Incorporated) in RPMI medium (Gibco BRL). Cells were incubated for 3 h at 37°C in a 5% CO_2_ atmosphere. Approximately 1.5×10^6^ cells were used for protein purification. Supernatant was removed and each well was washed twice with 1 mL of pre-warmed RPMI and incubated at 37°C for 10 min. Extracellular DNA formations were then digested with 10 U/ml DNase-1 (Fermentas, 798 Cromwell Park, USA) in 1 ml RPMI for 20 min. The activity of DNase-1 was blocked with 5 mM ethylenediaminetetraacetic acid (EDTA; Applichem, GmbH, Stockholm, Sweden). Samples were sequentially centrifuged at 300xg to remove whole cells and at 16000xg to remove cellular debris.

### Protein precipitation

Proteins were precipitated with cold acetone from identical volumes of culture supernatant. Samples were incubated overnight at −20°C and then centrifuged at 10.000xg for 15 min at 4°C. The pelleted proteins were lysed in lysis buffer (1% Triton X-100 and 150 mM NaCl in 20 mM HEPES pH 7.5) with protease inhibitors (Complete Protease Inhibitor Tablets; Roche).

### Western blot analysis

Western blot analysis was performed in cells treated for 45 min, as previously described [19]. Briefly, overnight incubation of the PVDF membranes was carried out at 4°C using an anti-LC3B polyclonal Ab (1/1000 dilution). Membranes were probed with HRP-conjugated secondary antibody (1/2000 dilution) for 45 min at room temperature. To verify equal loading, membranes were re-probed for GAPDH. Moreover, HMGB1-specific mouse mAb (1/1000 dilution) was utilized for the measurement of HMGB1 expressed in NETs, using the same protocol.

### Statistical analysis

Data are presented as means ± SD. Statistical analyses were performed using unpaired and paired t-test (n<6) and Wilcoxon paired test (n≥6). All statistical analyses were performed with GraphPad Prism (GraphPad Software, Inc.). *P* values of ≤0.05 were considered significant.

## Supporting Information

Figure S1 **Effect of treatment with PP1 or LY294002 on the release of NETs from PMNs from control subjects treated with MSU crystals for 180 min, as assessed by immunofluorescence microscopy.** DNA is labeled with DAPI (blue) and MPO is stained with anti-MPO mAb (green). Original magnification 400x. One out of four independent experiments is shown.
(TIF)

Figure S2 **Scatter plot showing the percentage of NET releasing synovial cells derived from six patients with gout compared to untreated control PMNs.**
(TIF)

Figure S3 A. Cytology. Isolated cells from synovial fluid from a patient with acute gout demonstrating the prevalence of neutrophils. Original magnification 1000x. Staining with May**-**Grumvald-Giemsa. B. Flow cytometric analysis of cell viability, using propidium iodide and Annexin-V staining, in untreated (CONTROL) and MSU treated (MSU) control neutrophils after 3 h of incubation. One out of three independent experiments is shown.
(TIF)
