# Supplementary figures and images for: Neutrophil Extracellular Trap Formation Is Associated with IL-1β and Autophagy-Related Signaling in Gout
Source: PLoS One. 2011 Dec 16;6(12):e29318. doi: 10.1371/journal.pone.0029318 (PMC3241704; doi:10.1371/journal.pone.0029318)

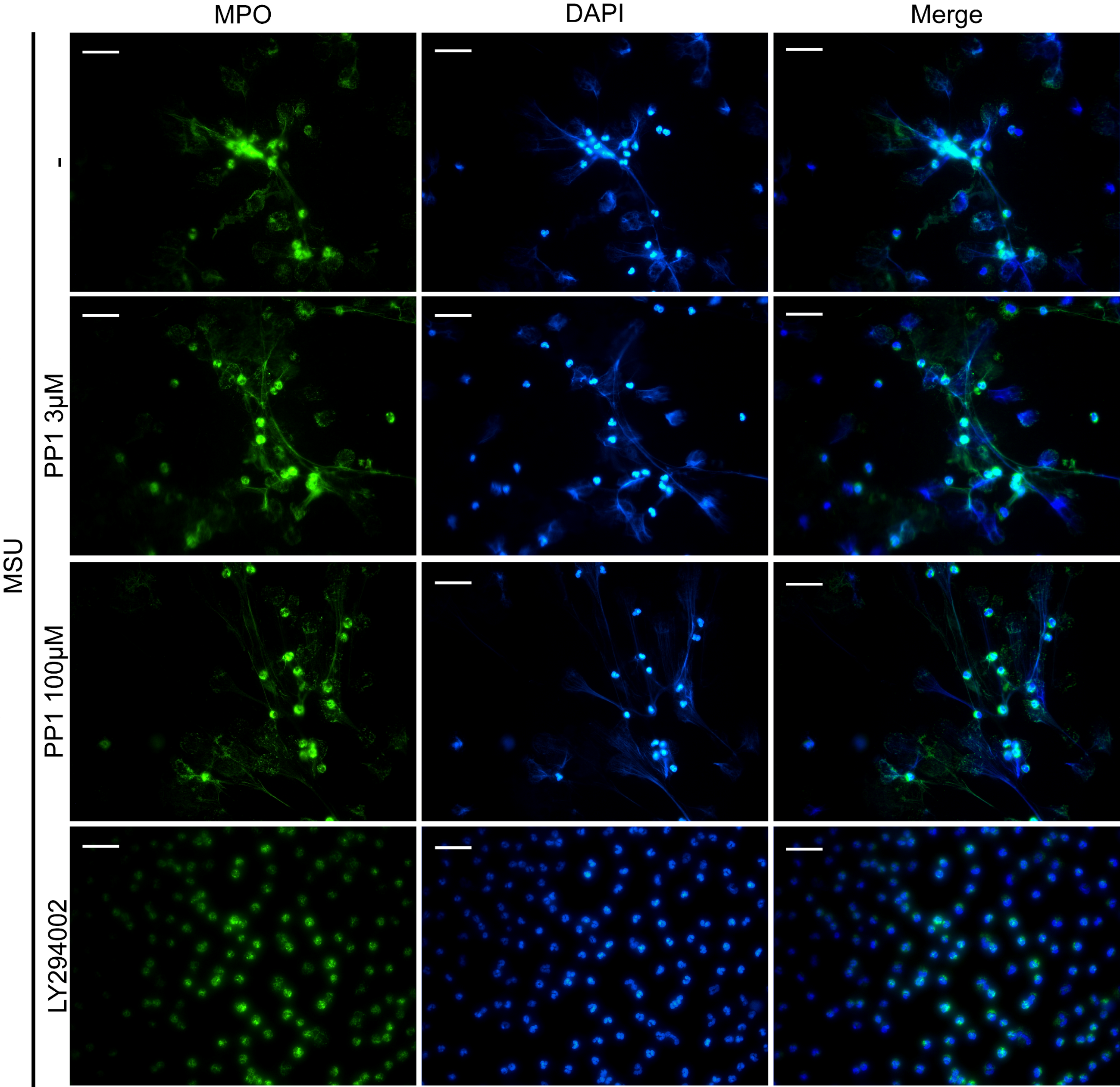

Supplement: Figure S1 — Effect of treatment with PP1 or LY294002 on the release of NETs from PMNs from control subjects treated with MSU crystals for 180 min, as assessed by immunofluorescence microscopy. DNA is labeled with DAPI (blue) and MPO is stained with anti-MPO mAb (green). Original magnification 400x. One out of four independent experiments is shown. (TIF) [file pone.0029318.s001.tif]

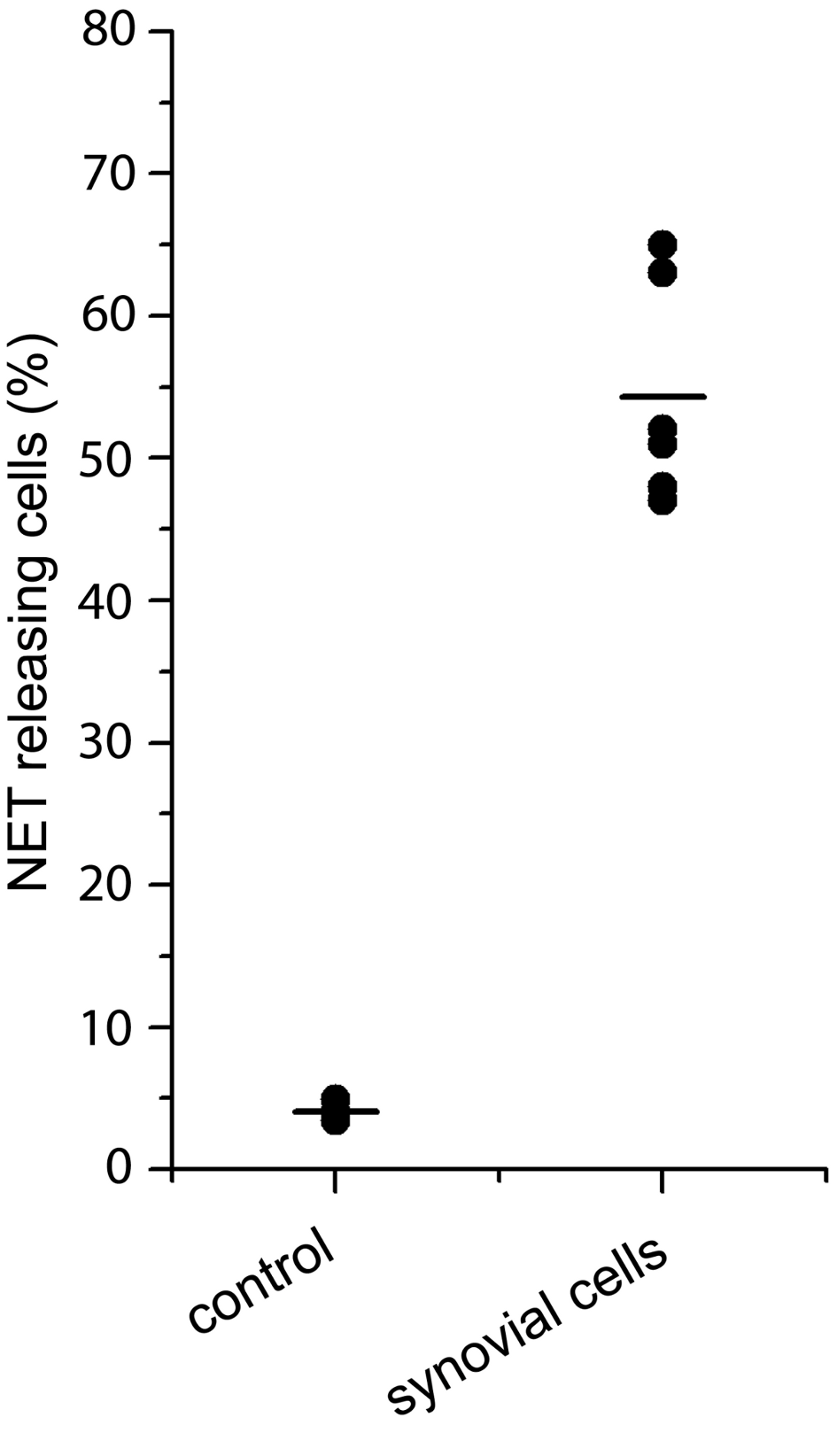

Supplement: Figure S2 — Scatter plot showing the percentage of NET releasing synovial cells derived from six patients with gout compared to untreated control PMNs. (TIF) [file pone.0029318.s002.tif]

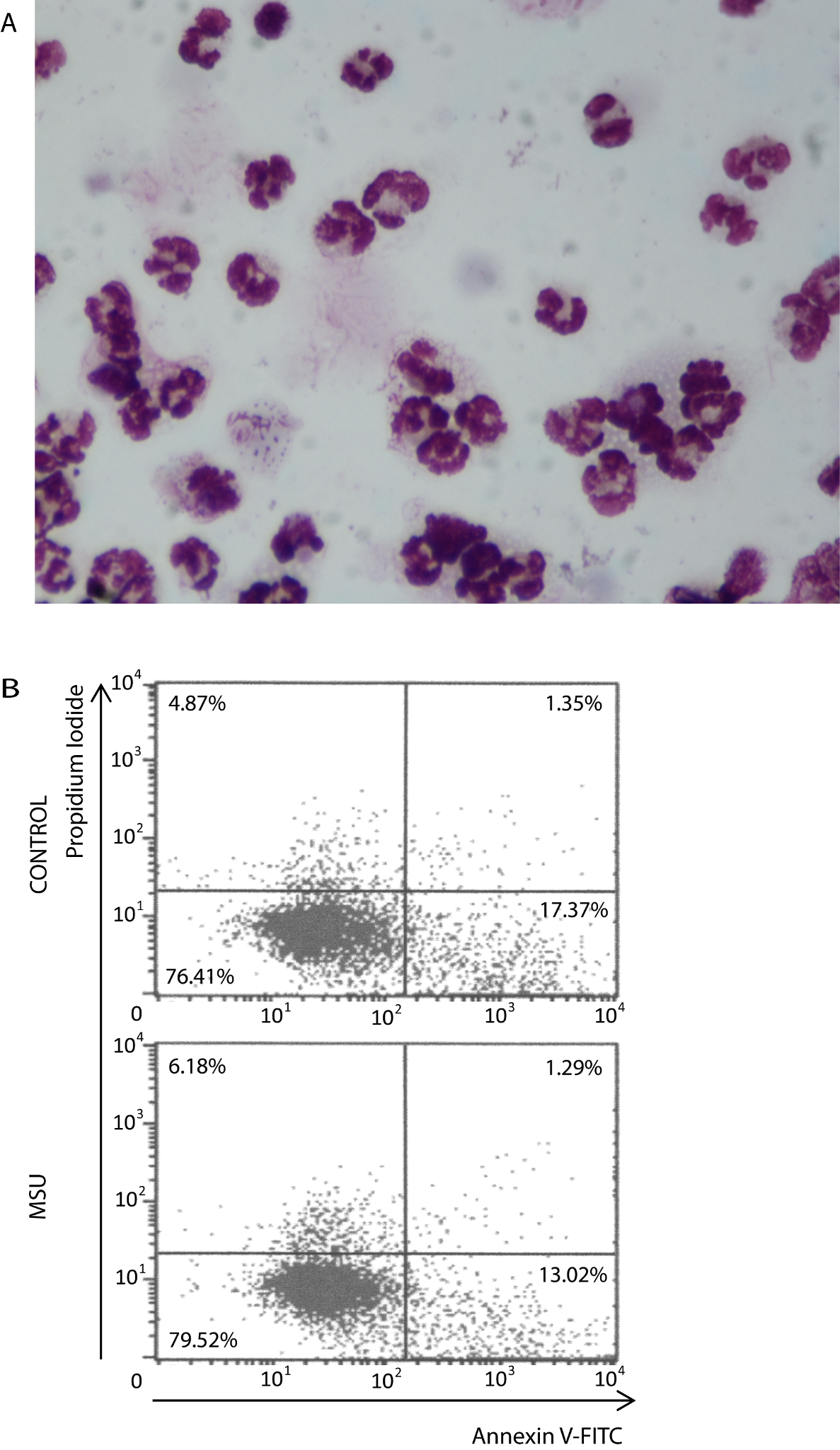

Supplement: Figure S3 — A. Cytology. Isolated cells from synovial fluid from a patient with acute gout demonstrating the prevalence of neutrophils. Original magnification 1000x. Staining with May-Grumvald-Giemsa. B. Flow cytometric analysis of cell viability, using propidium iodide and Annexin-V staining, in untreated (CONTROL) and MSU treated (MSU) control neutrophils after 3 h of incubation. One out of three independent experiments is shown. (TIF) [file pone.0029318.s003.tif]
